# Investigation of endoplasmic reticulum stress parameters in patients with gestational diabetes mellitus: A prospective study

**DOI:** 10.1002/ijgo.70091

**Published:** 2025-03-27

**Authors:** Sevcan Sarıkaya, Eissa Almaghrebi, Fatma Akat, Eda Nur Hepbildi, Esra Kosucu, Muslu Kazım Körez, Husamettin Vatansev

**Affiliations:** ^1^ Department of Obstetrics and Gynecology Health Sciences University, Konya City Hospital Konya Türkiye; ^2^ Department of Medical Biochemistry Selcuk University Faculty of Medicine Konya Türkiye; ^3^ Department of Medical Biostatistics Selcuk University Faculty of Medicine Konya Türkiye

**Keywords:** activating transcription factor‐6, biomarker discovery, C/EBP‐homologous protein, endoplasmic reticulum stress biomarkers, gestational diabetes mellitus, inositol‐requiring enzyme 1

## Abstract

**Objective:**

Our aim in this study was to evaluate the levels of Activating Transcription Factor‐6 (ATF‐6), Inositol‐Requiring Enzyme 1 (IRE‐1), and C/EBP‐Homologous Protein (CHOP), which are critical markers of endoplasmic reticulum (ER) stress, in patients with gestational diabetes mellitus (GDM), a condition increasingly recognized for its complex biochemical and molecular underpinnings.

**Materials and Methods:**

A total of 89 patients aged 18–45 years who applied to our hospital between January 2024 and December 2024 were included in our study. Of these, 45 patients were diagnosed with GDM during their routine pregnancy follow‐up, while 44 were healthy pregnant women without any additional metabolic or obstetric complications. Serum samples were analyzed for ATF6, IRE‐1, and CHOP levels, which are biochemical markers associated with ER stress. The data obtained were compared between the two groups.

**Results:**

The patients with GDM were older than those in the control group (31.98 ± 5.29 vs. 27.55 ± 5.92 years, *P* < 0.001). The mean number of gravidas (3.96 ± 2.01 vs. 2.48 ± 1.62, *P* < 0.001) and parity (2.18 ± 1.45 vs. 1.14 ± 1.07, *P* < 0.001) were significantly higher in the GDM group than in the control group. All GDM patients showed elevated levels of hemoglobin A1c (HbA1C) (6.69 ± 1.43% or 49.24 ± 15.25 mmol/mol, *n* = 45), indicating chronic hyperglycemia. GDM patients had elevated levels of red blood cells (RBC), hematocrit (HCT), mean platelet volume (MPV), platelet distribution width (PDW), red cell distribution width‐coefficient of variation (RDW‐CV) and potassium. They also had decreased glucose levels. Analysis revealed that there was no significant change in CHOP and IRE‐1 levels in the GDM women compared with patients in the control group. However, the level of ATF‐6 was significantly reduced in the GDM group compared with the control group (5.30, interquartile range [IQR] 4.59–6.28 vs. 5.92, IQR 5.17–8.43; *P* = 0.040). Despite no significant changes in CHOP and IRE‐1 levels, the reduced ATF‐6 levels in GDM patients highlight its potential as a specific biomarker for ER stress in the context of GDM.

**Conclusion:**

This study identifies ATF‐6 as a potential biomarker for GDM, with reduced levels significantly associated with GDM and key clinical parameters. Our findings support the integration of ER stress and hematological markers into future research and clinical practice for GDM.

## 1 INTRODUCTION

Gestational diabetes mellitus (GDM) is one of the most prevalent complications of pregnancy, affecting an estimated 5%–20% of pregnancies worldwide, depending on diagnostic criteria and population characteristics.^1^, ^2^ GDM, which is characterized by glucose intolerance initially identified during pregnancy, carries a number of serious hazards, such as pre‐eclampsia, fetal macrosomia, and neonatal hypoglycemia, in addition to long‐term effects such mother type 2 diabetes mellitus and offspring obesity.^3^, ^4^ Despite these risks, the precise pathophysiology of GDM remains incompletely understood, limiting advancements in diagnostic and therapeutic strategies.

The role of endoplasmic reticulum (ER) stress in metabolic disorders has emerged as a critical area of research. ER stress activates the unfolded protein response (UPR), which aims to restore cellular homeostasis but may trigger pathological pathways when dysregulated. Major signaling pathways involved in the UPR include Activating Transcription Factor‐6 (ATF‐6), Inositol‐Requiring Enzyme 1 (IRE‐1), and C/EBP‐Homologous Protein (CHOP). ATF6 is an ER membrane protein that, under conditions of ER stress, undergoes a process of translocation to the Golgi apparatus, where it is cleaved and activated. The cleaved ATF6 subsequently migrates to the nucleus, where it activates the transcription of genes involved in protein folding and the stress response. Its dysregulation has been implicated in various metabolic disorders, including diabetes.^5^ IRE‐1 serves as a pivotal sensor of ER stress, a condition that arises when the cell is unable to fold proteins properly. Upon activation, IRE‐1 initiates a signaling cascade involving the splicing of X‐box‐binding protein 1 (XBP1) mRNA, thereby enhancing the cell's capacity to manage misfolded proteins. However, excessive activation of IRE‐1 has been associated with β‐cell dysfunction and insulin resistance in metabolic diseases.^6^ CHOP is a transcription factor that plays a pivotal role in ER stress‐induced apoptosis. In the context of prolonged or unresolved ER stress, CHOP has been observed to promote cell death by downregulating anti‐apoptotic proteins, such as Bcl‐2, and upregulating pro‐apoptotic factors. This disruption of cellular homeostasis contributes to the initiation of the apoptotic process.^5^, ^6^ These markers have been implicated in insulin resistance, chronic inflammation, and beta‐cell dysfunction in non‐pregnant populations with metabolic syndromes.^5^, ^6^, ^7^ However, their relevance in GDM, particularly in comparison to healthy pregnancies, remains underexplored. In addition to ER stress markers, several hematological parameters such as mean platelet volume (MPV), platelet distribution width (PDW), and red cell distribution width (RDW) have been associated with systemic inflammation and metabolic dysregulation.^8^, ^9^

Prolonged ER stress and elevated ATF6 expression have also been linked to the development of diabetes mellitus (DM) as a result of pancreatic beta cell death. ATF6 plays a crucial role in the transcription of the protein folding machinery through the molecular chaperones.^10^, ^11^ The transmembrane protein kinase/endoribonuclease known as IRE‐1 is found in the ER and is triggered by ER stress. Dimerization, autophosphorylation, and IRE‐1 activation are the outcomes of the IRE‐1–binding immunoglobulin protein (BiP) complex's detection of unfolded proteins in the ER.^12^, ^13^ It has been demonstrated that reduced insulin mRNA expression in pancreatic β‐cells is linked to hyperactivation of IRE‐1. These findings have led to the hypothesis that IRE‐1 triggers endonucleolytic cleavage of the mRNA encoding insulin, the primary secretory protein in pancreatic β‐cells, when ER folding capacity is surpassed.^14^

One of the most significant mediators of ER stress‐induced apoptosis, CHOP operates via a variety of pathways.^15^ CHOP causes the BCL‐2 family members involved in cell survival to be down‐regulated and the upPro‐apoptotic Bcl‐2 homology 3 (BH‐3)‐only proteins that are essential for mitochondrial‐dependent apoptosis are regulated.^16^ Furthermore, reactive oxygen species generation and calcium release from the ER to the cytosol are caused by CHOP activation. The regulation of the oxidative state is another way that CHOP works, as overexpression of CHOP exacerbates the rise in reactive oxygen species at the ER.^17^, ^18^

This study prospectively investigates the relationship between ATF‐6, IRE‐1, and CHOP levels and critical hematological and demographic characteristics in pregnant women with and without GDM. It aims to evaluate the diagnostic potential of these markers and their association with clinical characteristics, offering fresh perspectives on the etiology and treatment of GDM. The two groups' collected data were compared.

## 2 MATERIALS AND METHODS

### 2.1 Participants and sample collection

The present study is a prospective study that included a total of 89 patients aged 18–45 years who applied to our hospital between January 2024 and December 2024. Patients were given an oral glucose tolerance test during routine pregnancy follow‐ups between 24 and 28 weeks. A total of 45 patients with high test results and who were diagnosed with gestational diabetes and 44 healthy pregnant patients with test results within the normal range were included in the study. Those excluded from the study were: patients with chronic systemic diseases, rheumatological or hematological diseases, obstetric pathologies such as pre‐eclampsia and intrauterine growth retardation, patients with multiple pregnancies, patients with malignancy, and patients who used chronic medication outside of pregnancy, smoked, or used drugs. Serum samples were obtained from the participants using the remaining blood from the venous blood samples taken on an empty stomach for routine biochemistry tests. No additional invasive procedures were applied to the patients. Before being examined, the collected serum samples were centrifuged and kept at −80°C. Every participant in the research completed a written voluntary consent form (Table 1).

**TABLE 1:** Characteristics of the study population.

Parameters	Healthy pregnancy (*n* = 44)	Gestational diabetes (*n* = 45)	*P*‐value
**Demographic value**			
Age (years)	27.55 ± 5.92	31.98 ± 5.29	<0.001^a^
Height (cm)	162.84 ± 5.31	161.31 ± 5.97	0.224^a^
Weight (kg)	77.45 ± 12.35	83.28 ± 14.59	0.055^a^
BMI	29.32 ± 5.30	31.97 ± 5.11	0.023^a^
Gravida	2.48 ± 1.62	3.96 ± 2.01	<0.001^a^
Parity	1.14 ± 1.07	2.18 ± 1.45	<0.001^a^
Comorbidity	6 (13.6)	45 (100)	<0.001^b^
Pregnancy week	32.20 ± 1.71	32.18 ± 3.26	0.961^c^
**Hematologic parameters**			
HbA1C	—	6.69 ± 1.43	—
HbA1C (SI)	—	49.24 ± 15.25	—
WBC	9.35 (8.45–11.37)	8.97 (7.45–11.70)	0.640^d^
RBC	4.02 (3.82–4.32)	4.36 (3.92–4.61)	**0.006** ^d^
HGB	11.69 ± 1.12	12.00 ± 1.55	0.283^c^
HCT	35.12 ± 2.89	36.77 ± 4.05	**0.030** ^c^
MCV	87.05 ± 6.72	84.94 ± 6.68	0.140^a^
MCH	28.82 ± 2.83	27.76 ± 3.03	0.092^a^
MCHC	33.00 ± 1.20	32.58 ± 1.51	0.156^a^
PLT	231.43 ± 57.47	227.98 ± 62.10	0.786^a^
MPV	10.60 (10.28–11)	11.10 (10.45–11.65)	**0.028** ^d^
PCT	0.24 (0.21–0.26)	0.24 (0.21–0.31)	0.523^d^
NE count	7.09 (5.92–8.79)	6.26 (5.23–8.22)	0.188^d^
LY count	1.78 ± 0.52	1.92 ± 0.59	0.215^a^
MO count	0.61 ± 0.17	0.63 ± 0.20	0.699^a^
NE %	73.49 ± 5.80	71.59 ± 8.06	0.205^a^
LY %	18.80 ± 5.17	20.83 ± 6.99	0.123^a^
MO %	6.36 ± 1.44	6.61 ± 1.83	0.480^a^
PDW	12.35 (11.55–13.43)	13.60 (12.05–15.25)	0.**016** ^d^
RDW‐SD	43.63 ± 3.86	43.42 ± 5.37	0.832^a^
RDW‐CV	13.90 ± 1.05	14.51 ± 1.63	**0.040** ^c^
**Biochemical parameters**			
Glucose	81.5 (74.5–91)	13 (88–148)	<**0.001** ^d^
AST	16 (12–17.25)	17 (13–24)	0.095^d^
ALT	10 (8–12.25)	10 (8–13)	0.934^d^
Sodium	137.45 ± 2.32	137.53 ± 2.52	0.878^a^
Potassium	4.04 ± 0.26	4.28 ± 0.35	<**0.001** ^a^
Urine density	1.02 ± 0.01	1.02 ± 0.01	0.316^a^
pH	6.18 ± 0.67	6.13 ± 0.71	0.707^a^
Urine glucose positivity	1 (2.3)	6 (13.3)	0.110^e^
Ketone positivity	3 (6.8)	14 (31.1)	**0.008** ^b^
**ER stress parameters**			
CHOP	33.47 (29.65–42.69)	32.21 (29.75–36.02)	0.275^d^
ATF‐6	5.92 (5.17–8.43)	5.30 (4.59–6.28)	**0**.**040** ^d^
IRE‐1	283.85 (257.93–387.97)	286.46 (259.11–340.18)	0.656^d^

*Note*: Data were expressed as mean ± standard deviation or median (25th percentile–75th percentile) for numerical variables, and count (*n*) and percentage (%) for categorical variables.

Abbreviations: ALT, alanine aminotransferase; AST, aspartate aminotransferase; ATF‐6, activating transcription factor‐6; BMI, body mass index; CHOP, C/EBP‐homologous protein; HbA1C, hemoglobin A1c; HCT, hematocrit; HGB, hemoglobin; IRE‐1, inositol‐requiring enzyme 1; LY, lymphocyte; MCH, mean corpuscular hemoglobin; MCHC, mean corpuscular hemoglobin concentration; MCV, mean corpuscular volume; MO, monocyte; MPV, mean platelet volume; NE, neutrophil; PCT, plateletcrit; PDW, platelet distribution width; PLT, platelets; RBC, red blood cells; RDW‐CV, red cell distribution width‐coefficient of variation; RDW‐SD, red cell distribution width‐standard deviation; WBC, white blood cells.

^a^ Student's *t*‐test.

^b^ Yates continuity correction chi‐square test.

^c^ Welch's *t*‐test.

^d^ Mann–Whitney *U*‐test.

^e^ Fisher exact test.

### 2.2 Analysis of endoplasmic reticulum stress markers

Serum samples were analyzed for ATF6, IRE‐1, and CHOP levels, which are biochemical markers associated with ER stress. Analyses were carried out using commercially available enzyme‐linked immunosorbent assay (ELISA) kits (BT Lab, Zhejiang, China; ATF6, E7169Hu; IRE‐1; E6871Hu; CHOP, E7168Hu) in accordance with the manufacturer's instructions. Optical density (OD) values were measured with a CLARIOstar microtiter plate reader (BMG LABTECH, Offenburg, Germany) at a wavelength of 450 nm. The measurement results were converted to protein concentrations (pg/mL or ng/mL) using standard curves. The data obtained were compared between the two groups.

### 2.3 Statistical analysis

Statistical analysis was performed using R version 4.2.1 (www.r‐project.org). Numerical data were presented as mean ± standard deviation or median with quartiles (1st quartile to 3rd quartile), and the differences in these parameters between GDM and control groups were compared with Student's *t*‐test and Welch's *t*‐test and Mann–Whitney *U*‐test, as appropriate. Shapiro–Wilk's normality test and Levene's test was conducted to check the normality of the data and homogeneity of the variances, respectively.

The Yates continuity correction chi‐square test and Fisher exact test were used to compare the relationship between the study groups and the categorical variables, which were characterized as number (*n*) and percentage (%). Spearman's rho correlation analysis was run to evaluate the relationship between ATF‐6 levels and numerical parameters. The receiver operating characteristic (ROC) curve was used to determine the diagnostic value of ATF‐6 level for predicting of GDM. Youden index criteria was used to determine the optimal cut‐off point. Area under the curve (AUC) was calculated with 95% confidence interval (CI) for the corresponding cut‐off point. Sensitivity and specificity values were obtained for this value. Results were considered significant at *P* < 0.05.

## 3 RESULTS

The patients with GDM were older than those in the control group (31.98 ± 5.29 vs. 27.55 ± 5.92, *P* < 0.001). The mean number of gravidas (3.96 ± 2.01 vs. 2.48 ± 1.62, *P* < 0.001) and parity (2.18 ± 1.45 vs. 1.14 ± 1.07, *P* < 0.001) were significantly higher in the GDM group than in the control group, whereas mean heights and weights were not significantly different. All GDM patients showed elevated levels of hemoglobin A1c (HbA1C) (6.69 ± 1.43% or 49.24 ± 15.25 mmol/mol, *n* = 45), indicating chronic hyperglycemia. GDM patients had elevated levels of red blood cells (RBC), hematocrit (HCT), MPV, PDW, RDW‐coefficient of variation (RDW‐CV), and potassium. They also had decreased glucose levels.

Comparing the GDM women with patients in the control group, analysis showed no discernible change in CHOP and IRE‐1 levels. In contrast to the control group, the GDM group's ATF‐6 level was considerably lower (5.30 [IQR 4.59–6.28] vs. 5.92 [IQR 5.17–8.43], *P* = 0.040).

**FIGURE 1:**
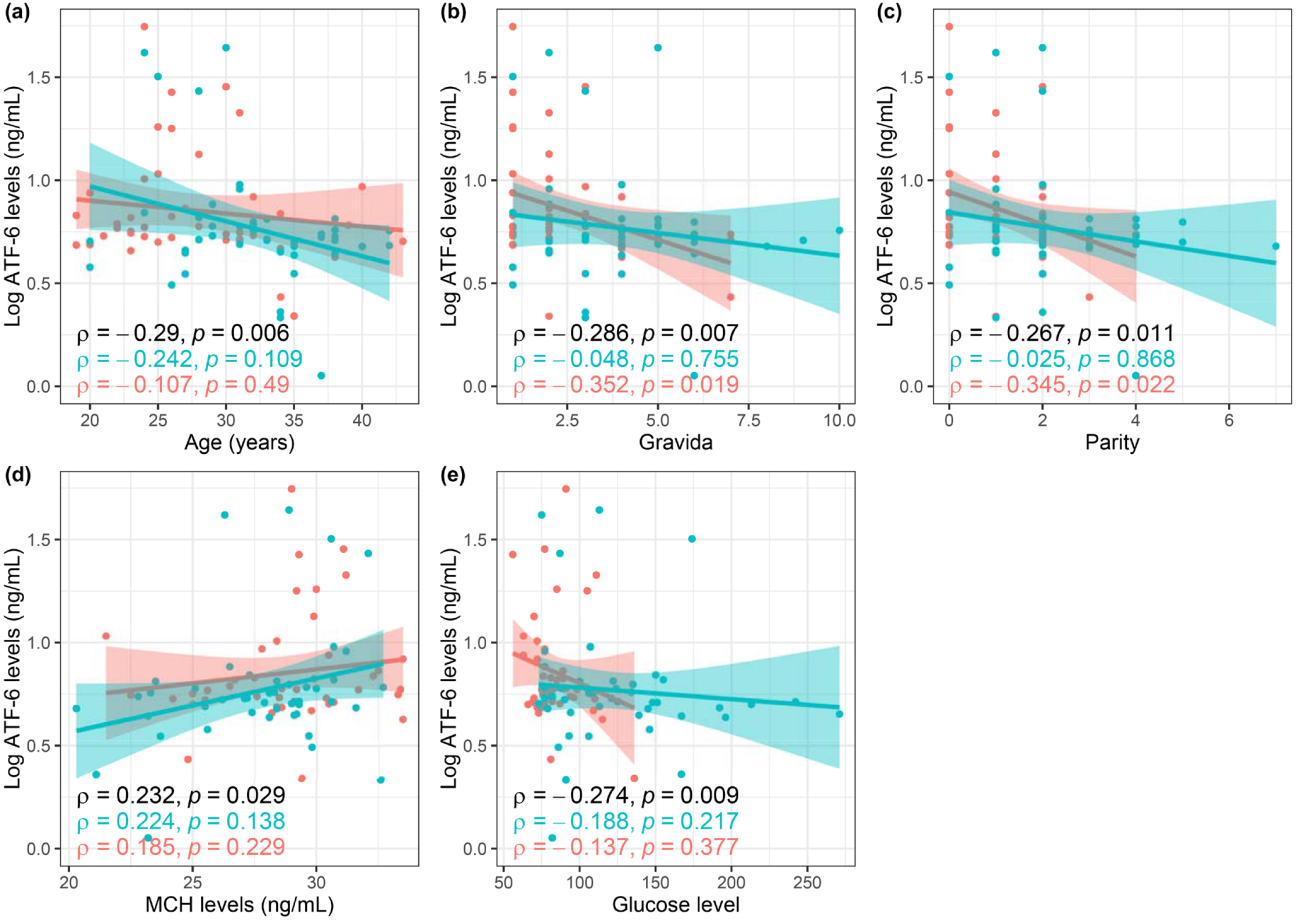
Correlation plot. (a–e) The scatter plots between logarithmic Activating Transcription Factor‐6 (ATF‐6) levels and age (a), gravida (b), parity (c), mean corpuscular hemoglobin (MCH) level (d), and glucose level (e), both overall and stratified by study groups. Black, depicts the relationship in overall patients; green depicts the relationship in gestational diabetes mellitus (GDM) patients; and red depicts the relationship in healthy controls.

In correlation analysis, a negative and significant relationship was found between ATF‐6 levels and age (Spearman's rho = −0.290, *P* = 0.006), gravida (Spearman's rho = −0.286, *P* = 0.007) and parity (Spearman's rho = −0.267, *P* = 0.011). Furthermore, lower glucose level (Spearman's rho = −0.274, *P* = 0.009) and elevated mean corpuscular hemoglobin (MCH) level (Spearman's rho = 0.232, *P* = 0.029) were correlated with increased ATF‐6 level (Figure 1).

**FIGURE 2:**
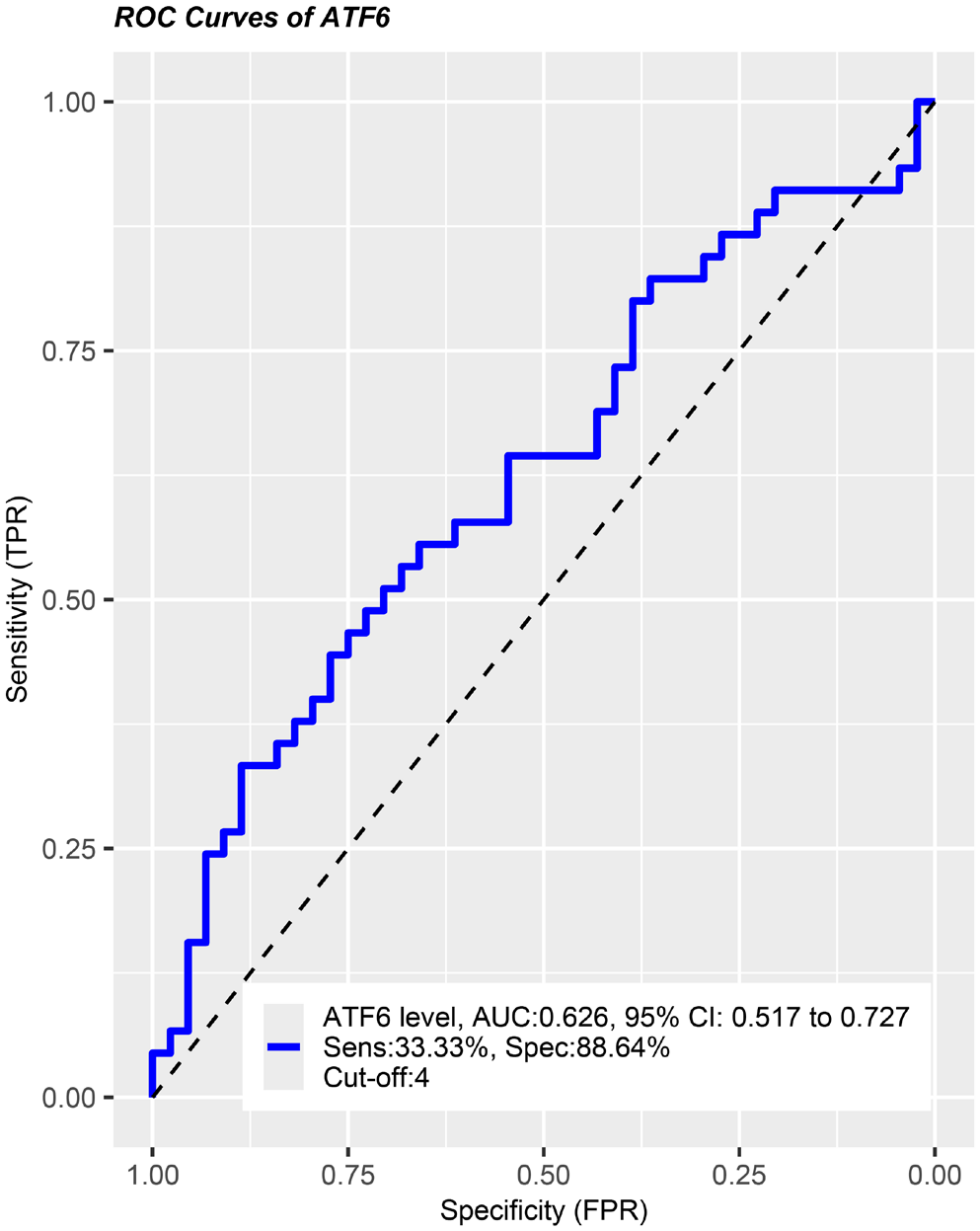
Receiver operating characteristic (ROC) curve for Activating Transcription Factor‐6 (ATF‐6) levels for discriminating gestational diabetes mellitus (GDM) patients from controls. The optimal cut‐off value was obtained by Youden *J*‐index. (*J =* max[sensitivity + specificity – 1]). CI, confidence interval.

Figure 2 demonstrated the ROC curve for ATF‐6 levels for discriminating GDM from controls. The optimal cut‐off value was 4.834, and the AUC was 0.626 (95% CI 0.517–0.727; *P* = 0.035). The sensitivity and specificity of the ATF‐6 for the corresponding cut‐off point were 33.33% and 88.64%, respectively.

## 4 DISCUSSION

This study prospectively evaluated the levels of ER stress markers (ATF‐6, IRE‐1, and CHOP) alongside hematological and demographic parameters in pregnant women with GDM compared with healthy controls. The findings revealed a significant reduction in ATF‐6 levels in the GDM group, while IRE‐1 and CHOP levels showed no significant differences. ATF‐6 levels were negatively correlated with age, gravidity, parity, and glucose levels, suggesting its potential role in the pathophysiology of GDM. Additionally, GDM was associated with significant differences in hematological markers, including elevated MPV, PDW, RDW, and potassium levels, as well as higher gravidity and parity compared with controls. These results highlight the multifaceted nature of GDM‐related metabolic and systemic changes.

This study has certain limitations. Although the prospective design strengthens the causal interpretation of findings, the moderate sample size and single‐center data collection may limit generalizability. Furthermore, the observed changes in ER stress markers and hematological parameters represent associations, and mechanistic studies are needed to elucidate their causal roles in GDM pathogenesis. Additionally, while ATF‐6 demonstrated moderate diagnostic potential with high specificity, its sensitivity was limited, underscoring the need for multimarker approaches in clinical practice.

Numerous physiological processes, such as cell differentiation and survival/apoptosis, migration, invasion, and angiogenesis, are mediated by ER stress/UPR signaling.^19^ Additionally, it has been proposed that ER stress has a role in low birth weight, pre‐eclampsia, recurrent pregnancy loss, and fetal growth limitation.^20^, ^21^, ^22^ Vascular endothelial growth factor also regulates the expression of the UPR‐related proteins IRE1α, PERK and ATF6, which are essential for proper placental vascular development. The UPR‐related proteins IRE‐1α, PERK, and ATF6 regulate an expression that is essential for proper placenta vascular development.^23^ To the best of our knowledge, our study is the first to show the relationship between GDM and ER stress parameters.

Comparing our findings with the existing literature reveals both consistencies and divergences. Elevated ER stress markers have been reported in non‐pregnant populations with metabolic syndromes, including type 2 diabetes.^5^, ^6^ ER activity is also linked to signaling networks that regulate the metabolic destiny of nutrients such as glucose and amino acids. During the UPR, transcriptional regulation of glucose synthesis or breakdown pathways occurs, and the ER is very sensitive to changes in energy and glucose availability.^24^, ^25^ Through a controlled interaction with CRTC2 (CREB‐regulated transcription coactivator 2, also known as TORC2), ATF6 has recently been linked to the regulation of hepatic glucose synthesis.^26^ By interfering with the CREB‐CRTC2 connection and preventing CRTC2 occupancy at the promoters of genes implicated in gluconeogenesis, ATF6 activation may lower hepatic glucose production. Given that overexpression of ATF6 in the livers of obese animals seems advantageous in reversing the effects of CRTC2 on the gluconeogenic program, this interaction between ATF6 and CRTC2 may be a significant factor in the impairment of hepatic gluconeogenesis in obesity and type 2 diabetes.^5^ In our study, a decrease in ATF‐6 levels was observed in GDM patients. However, the observed reduction in ATF‐6 levels in GDM suggests pregnancy‐specific adaptations in UPR signaling. It is plausible that the reduced ATF‐6 levels reflect a compensatory mechanism aimed at minimizing systemic inflammation during pregnancy, a hypothesis supported by studies demonstrating gestational modulation of immune and metabolic pathways.^10^, ^11^ Although ATF‐6 exhibited high specificity (88.64%), its sensitivity (33.33%) was comparatively low, thereby constraining its utility as a standalone diagnostic marker for GDM. This finding indicates that ATF‐6 should not be utilized in isolation, but rather as a component of a multimarker panel that incorporates additional biochemical and hematological parameters. Subsequent research endeavors should investigate the combination of ATF‐6 with supplementary markers to augment the overall predictive value in clinical settings.

Pancreatic β‐cells, which are responsible for insulin production, are highly sensitive to ER stress due to their substantial protein synthesis demands. Activation of IRE‐1α in response to elevated glucose levels initiates the splicing of XBP1 mRNA, leading to the production of a transcription factor that enhances the expression of genes involved in protein folding and secretion. This process is essential for maintaining β‐cell function and viability. Disruption of the IRE‐1α/XBP1 pathway impairs proinsulin synthesis and increases oxidative stress, contributing to β‐cell dysfunction and the development of diabetes.^27^, ^28^ Beyond its role in β‐cells, IRE‐1α influences insulin sensitivity in peripheral tissues. Chronic ER stress, often associated with obesity, leads to sustained IRE‐1α activation, which can impair insulin signaling pathways. In diet‐induced obese mice, pharmacological suppression of IRE‐1α's RNase activity has been demonstrated to increase insulin sensitivity and glucose tolerance, indicating that IRE‐1α activity modulation may be a therapeutic approach for improving insulin responsiveness.^28^ IRE‐1α also plays a role in the complications associated with diabetes. For instance, in diabetic wound healing, IRE‐1α modulates microRNAs that affect bone marrow progenitor cell function, thereby influencing angiogenesis and tissue repair. Overexpression of IRE‐1α in these cells has been shown to improve wound healing in diabetic models, highlighting its potential in mitigating diabetes‐related complications.^28^ Placental tissues from GDM and healthy pregnancies showed elevated markers of ER stress, including CHOP, in GDM cases. These findings suggest that while ER stress is a normal adaptive response in healthy pregnancies, its dysregulation in GDM contributes to adverse outcomes, including β‐cell dysfunction and insulin resistance.^29^ However, in the present study, no statistically significant differences were detected between GDM and healthy pregnancies in terms of IRE‐1 and CHOP levels. In contrast, the lack of significant changes in IRE‐1 and CHOP levels aligns with reports indicating that UPR activation is pathway‐specific and context‐dependent.^7^, ^12^ Although ATF‐6 demonstrated a significant reduction in GDM patients, CHOP and IRE‐1 levels did not show significant differences, suggesting a potential context‐dependent activation of the UPR pathways. Previous studies have indicated that ATF‐6 plays a pivotal role in early ER stress response, while CHOP and IRE‐1 activation may be more pronounced in chronic or severe ER stress conditions. Furthermore, pregnancy is a unique physiological state marked by systemic adaptations that may modulate ER stress responses in a manner distinct from that observed in non‐pregnant metabolic disorders. The findings underscore the necessity for additional research to elucidate the specific temporal and mechanistic roles of these markers in the pathophysiology of GDM.

The observed reduction in ATF‐6 levels in GDM patients suggests a potential compensatory mechanism aimed at mitigating systemic inflammation during pregnancy. Previous studies have indicated that ATF‐6 plays a crucial role in β‐cell homeostasis and insulin secretion, with its regulation varying in response to chronic hyperglycemia.^10^ Given that pregnancy involves distinct hormonal and immunomodulatory adaptations, it is plausible that ATF‐6 downregulation serves to counteract excessive metabolic stress.^20^ However, this hypothesis requires further validation. Future studies should investigate ATF‐6 expression dynamics across different stages of pregnancy, particularly in placental tissues and β‐cells, to determine whether its downregulation represents a transient adaptation or a pathological shift. Experimental studies utilizing cell culture and animal models could provide deeper insights into the interplay between ATF‐6 and pregnancy‐related inflammatory responses.^14^

The UPR is triggered by an accumulation of unfolded or misfolded proteins in the ER lumen, which results in ER stress. CHOP is a key mediator of the UPR and is upregulated during prolonged ER stress, leading to apoptosis. In the context of metabolic diseases, CHOP has been implicated in the dysfunction of pancreatic β‐cells and the development of insulin resistance. For instance, studies have shown that deletion of the CHOP gene in mice improves β‐cell function and enhances glycemic control, despite the presence of obesity.^30^, ^31^

Gestational diabetes mellitus is characterized by glucose intolerance with onset or first recognition during pregnancy. The physiological insulin resistance that develops during pregnancy is typically compensated by increased insulin secretion. However, in GDM, this compensatory mechanism is inadequate, leading to hyperglycemia. While direct studies linking CHOP to GDM are limited, it is plausible that ER stress and subsequent CHOP‐mediated apoptosis in pancreatic β‐cells could contribute to the impaired insulin secretion observed in GDM.^32^

While ATF‐6 levels were significantly lower in GDM patients, CHOP and IRE‐1 levels did not demonstrate significant changes. This finding may be explained by the dynamic and context‐dependent activation of UPR pathways. CHOP has been predominantly linked to prolonged and severe ER stress, which can result in apoptosis. In contrast, IRE‐1 activation has been associated with both adaptive and maladaptive responses to cellular stress.^15^ The absence of significant changes in CHOP and IRE‐1 levels suggests that the degree of ER stress in GDM patients may not be severe enough to trigger apoptosis‐driven pathways or that compensatory mechanisms maintain cellular homeostasis.^20^

The temporal aspect of sample collection can also influence the observed marker levels. CHOP and IRE‐1 activation may be transient or dependent on specific metabolic conditions, and a single time‐point measurement may not capture fluctuations in their expression. Consequently, prospective studies should investigate the temporal progression of these markers during distinct phases of pregnancy to ascertain whether CHOP and IRE‐1 alterations transpire at subsequent stages of GDM progression. Furthermore, studies that utilize placental tissue or investigations focused on β‐cell‐specific functions could offer more profound insights into their roles in GDM pathophysiology.^10^

Mean platelet volume reflects the average size of platelets and serves as an indicator of platelet activation. Studies have reported elevated MPV levels in pregnant women with GDM compared with those with normal pregnancies. PDW measures the variability in platelet size and is another marker of platelet activation. Research indicates that PDW levels are elevated in GDM patients. RDW reflects the variation in red blood cell size and is commonly used to assess anemia. Recent studies have explored its association with GDM.^33^ In our study, MPV, PDW and RDW levels were found to be high in patients with GDM, which is consistent with the literature. The elevated MPV, PDW, and RDW observed in this study are consistent with prior findings linking these markers to inflammation and vascular dysfunction in metabolic disorders, further supporting their relevance in GDM.^8^, ^13^

The implications of these findings are significant. ATF‐6 and associated hematological markers could serve as components of a multimarker panel for GDM screening, complementing established diagnostic criteria. The high specificity of ATF‐6, in particular, highlights its potential utility in identifying women at risk of GDM‐related complications. Clinically, these markers could aid in stratifying patients for targeted interventions, such as lifestyle modifications or pharmacological therapies. Future research should focus on large‐scale, multicenter studies to validate these findings and explore the integration of ER stress and hematological markers into personalized care pathways. Mechanistic studies are also needed to clarify the biological underpinnings of observed marker changes and their relevance to GDM progression.

Although ATF‐6 has exhibited promise as a biomarker for GDM, its comparatively low sensitivity (33.33%) curtails its efficacy as a standalone diagnostic instrument. To enhance diagnostic accuracy, future studies should explore the combination of ATF‐6 with additional biomarkers, including other ER stress markers (CHOP, IRE‐1), hematological parameters (MPV, RDW, PDW), and inflammatory markers (C‐reactive protein, tumor necrosis factor‐α, interleukin‐6). A multimarker panel approach has the potential to enhance both sensitivity and specificity, providing a more comprehensive assessment of GDM‐associated metabolic changes. Furthermore, the integration of machine learning algorithms to analyze biomarker combinations holds promise for offering more precise risk stratification and early detection of GDM. Conducting longitudinal studies that monitor these biomarkers across various pregnancy stages will be essential in ascertaining the most optimal combination for clinical implementation.

## 5 CONCLUSION

This study identifies ATF‐6 as a potential biomarker for GDM, with reduced levels significantly associated with GDM and key clinical parameters. Elevated hematological markers, including MPV, PDW, and RDW‐CV, further highlight the systemic changes in GDM. While ATF‐6 shows high specificity, its diagnostic utility requires validation in larger studies. These findings support the integration of ER stress and hematological markers into future research and clinical practice for GDM.

## AUTHOR CONTRIBUTIONS

SS and HV planned the study. SS, ENH, and EK performed the data collection. EA, FA, and HV analyzed the biochemical parameters. MKK performed the statistical analysis of the obtained data. All authors, especially SS, took part in the literature review and writing and reviewing of the article.

## FUNDING INFORMATION

The study received no funding from any institution or organization.

## CONFLICT OF INTEREST STATEMENT

The authors have no conflicts of interest.

## INFORMED CONSENT

Written informed consent was obtained from each patient who participated in the study.

## Data Availability

Research data are not shared.
